# Late onset Darier’s disease in a genetically predisposed individual: a case report

**DOI:** 10.11604/pamj.2022.42.208.32696

**Published:** 2022-07-15

**Authors:** Manoharan Dhanaraj, Geo Celestin Danny, Shreya Srinivasan, Sowmya Nagaraju

**Affiliations:** 1 Department of Dermatology, Venereology and Leprosy, Sree Balaji Medical College and Hospital, CLC Works Road, Chromepet, Chennai, Tamil Nadu 600044, India

**Keywords:** Darier’s disease, genodermatosis, dermoscopy, case report

## Abstract

Keratosis follicularis also called as Darier's disease, is a rare autosomal dominant cutaneous disease. It is characterized by greasy keratotic sometimes crusted red to brown papules and plaques over seborrheic areas and in flexures with nail abnormalities. It is well established that the disease begins between the ages of 6 and 20 years, with a peak onset during puberty. The disease tends to manifest early, especially with the family history of the disease. Hereby, we report a case of Darier's disease with a special interest in its late onset presentation despite having significant family history of the disease, along with clinicopathological and dermoscopic features. We also highlight the use of non-invasive investigative technique of dermoscopy as a tool to diagnose the disease.

## Introduction

Darier´s disease is an autosomal dominant genodermatosis caused by mutations in ATP2A2 gene. It involves seborrheic areas wherein hyperkeratotic papules and plaques can be seen [1]. Oral, laryngeal, esophageal, rectal mucosae are also less frequently involved [2]. Familial Darier´s disease is usually seen during puberty [3]. We hereby report a late onset case of Darier´s disease with clinicopathological and dermoscopic correlation.

## Patient and observation

**Patient information:** a 48-year-old male came to the dermatology out-patient department with complaints of multiple pigmented coalesced elevated lesions with few areas of normal skin in between, present over face, neck and upper chest for 6 months. Lesions first started over forehead and gradually progressed to involve cheeks, neck and upper chest. History of development of similar lesions over axillae and groin for 1 month. Patient complained of aggravation of lesions in summer. History of photosensitivity was present. Significant family history was present, wherein his mother, sister and brother had similar complaints with an earlier onset of the disease. History of frequent alcohol intake was also present.

**Figure 1:**
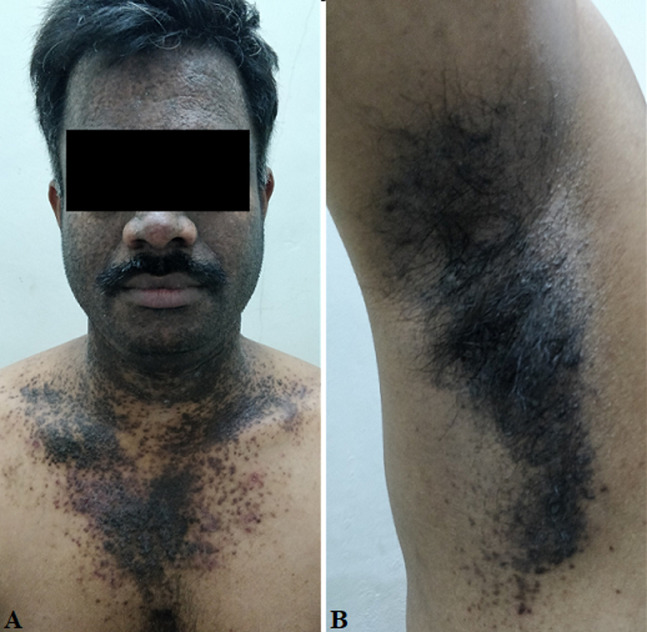
(A, B) clinical picture showing hyperpigmented papules and plaques over face, neck, upper chest and axilla

**Figure 2:**
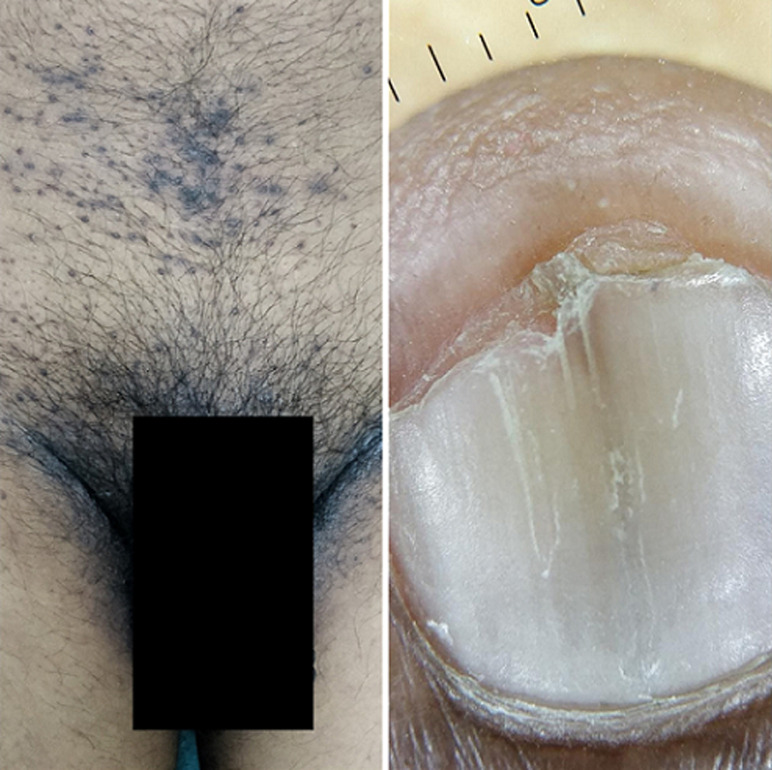
clinical picture showing hyperpigmented papules and plaques over groin and onychoscopy showing longitudinal striations and 'V' nicking of the right index finger nail (non-polarized mode, magnification 20X)

**Clinical findings:** multiple coalesced hyperpigmented papules and plaques were present over face, neck, upper chest, axillae and groin (Figure 1 (A, B), Figure 2). Oral mucosa, scalp, palms and soles were normal with no evidence of palmoplantar pitting. No systemic manifestations were seen. Radiological skeletal survey was done and association with multiple bone cysts was ruled out.

**Figure 3:**
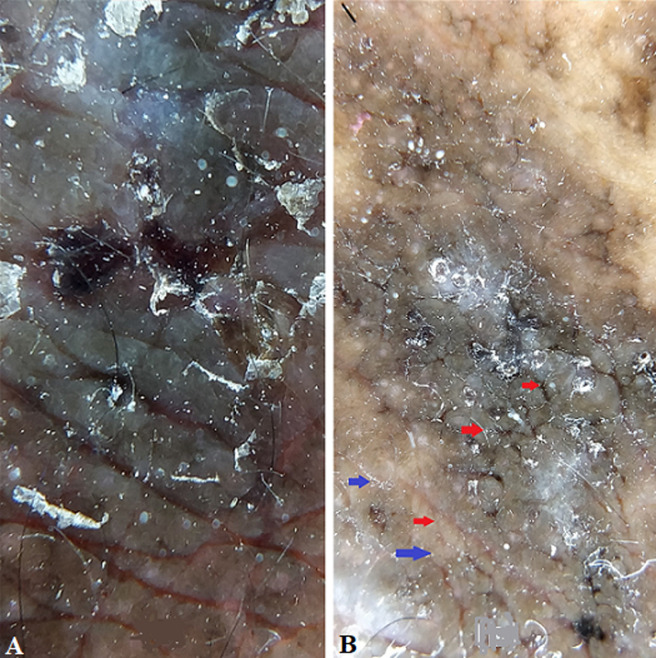
dermoscopic picture showing, (A) irregular linear parallel furrows with cracked riverbed-like appearance (polarized mode, magnification 20X); and (B) yellowish brownish areas with a white halo (blue arrow) and riverbed- like appearance (red arrows) (polarized mode, magnification 20X)

**Figure 4:**
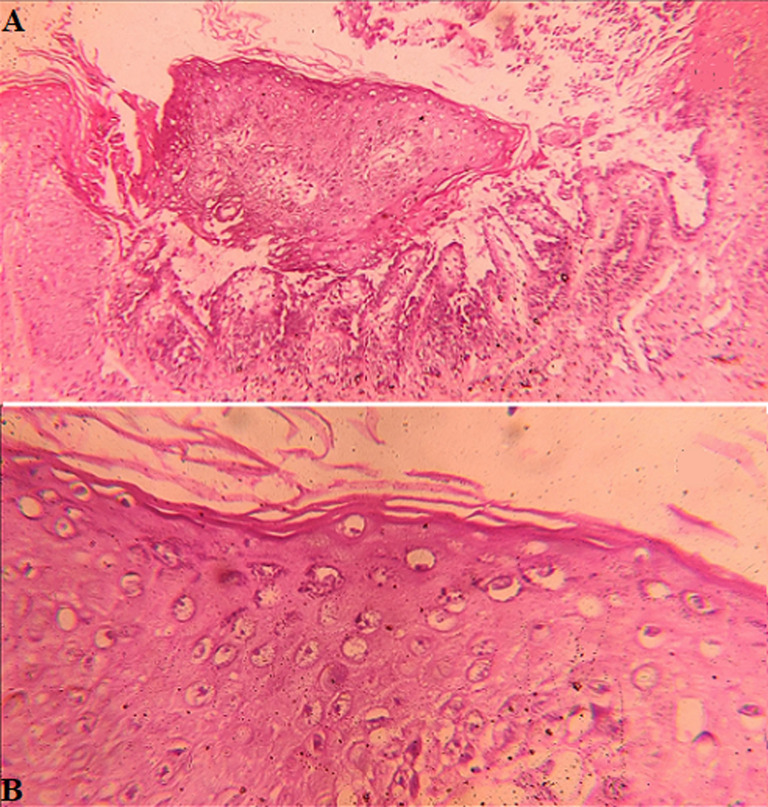
histopathological picture showing, (A) suprabasal cleft (scanner view, 10X); and (B) corps ronds (high power view, 40X)

**Figure 5:**
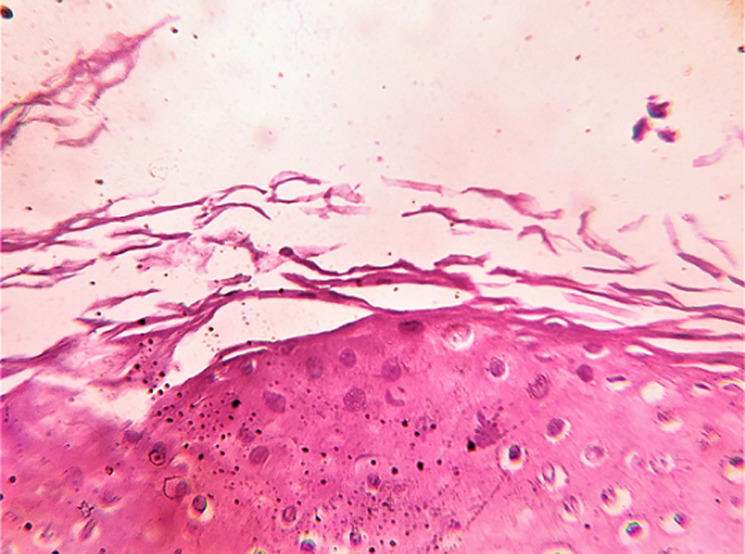
histopathological picture showing grains (high power view, 40X)

**Diagnostic assessment:** dermoscopy with hand held dermoscope and 20X magnification was performed on the lesions. Findings seen were irregular-linear parallel furrows with cracked riverbed-like appearance (Figure 3 A and red arrows in Figure 3 B) and yellowish brownish areas with a white halo (blue arrows in Figure 3 B). Onychoscopy with the same dermoscope was done which showed longitudinal striations and 'V' nicking of the right index finger nail distally (Figure 2). Histopathological examination was done from the punch biopsy taken from the lesion on the back which showed, suprabasal split (Figure 4 A), dyskeratotic keratinocytes- corps ronds in the malphigian layers of the epidermis (Figure 4 B) and grains (Figure 5).

**Diagnosis:** thus with the help of dermoscopic features and histopathology, a diagnosis of Darier´s disease was confirmed.

**Therapeutic interventions:** patient was advised oral retinoids along with general measures of sun protection by topical sunscreens.

**Follow-up and outcome of interventions:** the patient is still under follow up.

**Informed consent:** informed consent was provided by the patient.

## Discussion

Darier´s disease is a rare disorder of keratinization involving skin and less frequently the oral mucosa. It presents with keratotic papules and plaques in seborrheic regions and intertriginous areas with various nail abnormalities [4]. It usually manifests during childhood or adolescence and has an equal gender distribution. When there is history of Darier´s disease in the family, the onset of the disease tends to be earlier in life. Clinically, multiple erythematous small, firm papules appear first, later on become grayish brown, ulcerates and gets crusted and as the disease progresses, the hyperpigmentation increases. It may be associated with foul odor. Palmoplantar keratosis may be present consisting of fissuring, with nail changes like longitudinal streaking and subungual keratosis [5].

Histologically, supra basal clefts can be seen with dyskeratosis and acantholysis. Corps ronds have central pyknotic, homogenous, basophilic nuclei with a halo and are seen in upper malphigian layers. Grains can be seen in the stratum corneum and resemble large parakeratotic cells. The underlying dermal papillae project into these clefts and form villi-like structures. The lesion may be superimposed by a keratin plug showing parakeratosis. Dermoscopic findings of Darier’s disease include various patterns, most commonly a central yellowish to brownish area of polygonal to roundish or irregular shape, in addition to a whitish halo surrounding it. Parallel pink to reddish furrows having star-like, irregular, linear shapes can also be seen, indicative of acantholysis. These might be associated with scales or crusting which lead to a cracked riverbed-like appearance. Dermoscopy helps to analyze otherwise difficult to notice acral signs like palmar pits, acrokeratosis verruciformis like papules and punctate keratoderma.

The differential diagnosis include seborrheic dermatitis, Grover’s disease and Hailey-Hailey disease. Acral signs may mimic plane warts and acrokeratosis verruciformis of Hopf. Treatment mainly includes avoidance of triggering factors by sun protection, hygiene and loose clothing. The Mainstay of treatment involves the use of oral retinoids with topical retinoids playing a role in localized disease. Laser surgery, botulinum toxin, electrosurgery, dermabrasion constitute the procedural treatment.

## Conclusion

This case has been reported due to the late manifestation of Darier´s disease despite a positive family history of the same. Furthermore, studies in view of this should be done to elaborate the late onset of the disease. The case report also emphasizes the use of dermoscopy as a non invasive modality to screen for disease which can later be confirmed by histopathology.
